# A natural barrier to lateral gene transfer from prokaryotes to eukaryotes revealed from genomes: the 70 % rule

**DOI:** 10.1186/s12915-016-0315-9

**Published:** 2016-10-17

**Authors:** Chuan Ku, William F. Martin

**Affiliations:** Institute of Molecular Evolution, Heinrich-Heine University, Düsseldorf, Germany

## Abstract

**Background:**

The literature harbors many claims for lateral gene transfer (LGT) from prokaryotes to eukaryotes. Such claims are typically founded in analyses of genome sequences. It is undisputed that many genes entered the eukaryotic lineage via the origin of mitochondria and the origin of plastids. Claims for lineage-specific LGT to eukaryotes outside the context of organelle origins and claims of continuous LGT to eukaryotic lineages are more problematic. If eukaryotes acquire genes from prokaryotes continuously during evolution, then sequenced eukaryote genomes should harbor evidence for recent LGT, like prokaryotic genomes do.

**Results:**

Here we devise an approach to investigate 30,358 eukaryotic sequences in the context of 1,035,375 prokaryotic homologs among 2585 phylogenetic trees containing homologs from prokaryotes and eukaryotes. Prokaryote genomes reflect a continuous process of gene acquisition and inheritance, with abundant recent acquisitions showing 80–100 % amino acid sequence identity to their phylogenetic sister-group homologs from other phyla. By contrast, eukaryote genomes show no evidence for either continuous or recent gene acquisitions from prokaryotes. We find that, in general, genes in eukaryotic genomes that share ≥70 % amino acid identity to prokaryotic homologs are genome-specific; that is, they are not found outside individual genome assemblies.

**Conclusions:**

Our analyses indicate that eukaryotes do not acquire genes through continual LGT like prokaryotes do. We propose a 70 % rule: Coding sequences in eukaryotic genomes that share more than 70 % amino acid sequence identity to prokaryotic homologs are most likely assembly or annotation artifacts. The findings further uncover that the role of differential loss in eukaryote genome evolution has been vastly underestimated.

**Electronic supplementary material:**

The online version of this article (doi:10.1186/s12915-016-0315-9) contains supplementary material, which is available to authorized users.

## Background

Few topics in evolutionary biology have received as much attention in the last 20 years as lateral gene transfer (LGT, or horizontal gene transfer [HGT]) [1–3], with more than 11,000 papers that have appeared on the topic since 1985 and more than 30,000 citations to those papers in 2015 alone (Thomson Reuters Web of Science^TM^ as of 21 April 2016). Cognizant biologists have learned one thing for certain about LGT: Not all papers bearing claims for LGT are evidence for the workings of LGT, especially when it comes to LGT from prokaryotes to eukaryotes, which is the focus of our paper. For example, the original report of the human genome in 2001 [4] carried claims for hundreds of cases of prokaryote-to-eukaryote LGT in our own DNA. Those claims were, however, quickly unveiled as interpretation and annotation artifacts [5, 6]. More recently two papers on tardigrade genomes have provided a clear case in point: One report said that 16.1 % of the genes in the tardigrade genome were recently acquired via LGT from various prokaryotes [7], while an independent sequencing project stated that there was virtually no LGT in the tardigrade genome [8]. The main difference between the two studies was that in one study [7] genes probably belonging to associated bacteria were annotated as tardigrade genes. Those genes were not present in the other genome study [8], the scaffolds of which are longer, helping to filter out the contaminations that were interpreted as prokaryote-to-eukaryote LGT. Curiously, the claims for LGTs in the human genome, which were long ago refuted [5, 6], are now making their way back into the literature [9], based on analyses employing the same LGT identification software [10] used for the tardigrade genome that was reported to be LGT-rich [7]. Apart from the natural and well-documented process of gene acquisition from the ancestors of organelles in the wake of mitochondrial and plastid origin — endosymbiotic gene transfer [11, 12] — how much prokaryote-to-eukaryote LGT, if any, is really going on in nature?

Within the prokaryotes, LGT is best seen as a way of life. Several naturally occurring mechanisms of LGT among prokaryotes have been known for many decades: transfer by naked DNA uptake from the environment (transformation), transfer by plasmid transfer (conjugation), transfer via phage particles (transduction), and gene transfer agents [13–18]. A great deal is known about the genes and proteins that moderate these LGT mechanisms in prokaryotes [19–21]. These LGT mechanisms merely introduce DNA into the prokaryotic cell; whether or not it recombines into the genome is governed by the genes and proteins that mediate DNA insertion and/or recombination [22, 23].

Importantly, the mechanisms that introduce DNA into the cell for LGT are the same that introduce DNA into the cell for normal recombination within prokaryotic species [24]. In prokaryotes, recombination is never reciprocal. It is always unidirectional from donor to recipient, and with transformation, transduction, or gene transfer agents, the donor and recipient do not even need to ever physically meet. Prokaryotic genomes are highly dynamic in terms of gene content. They are typically replete with LGT, undergoing continuous gains (often from outside the species, genus, or family) and losses through deletion [2, 25–27]. Over time, these gains and losses lead to pangenome structures [12, 28–30], not only at the species level but at all taxonomic levels [12]. In prokaryotes, acquisition through LGT dwarfs the role of gene duplication in generating gene families within genomes [31]. Prokaryotic LGT is pivotal in the spread of antibiotic resistance [32] and in ecological adaptation [33]. The existence and extent of LGT in prokaryotes has challenged the traditional view of prokaryotic evolution as a fundamentally tree-like process and has prompted the use of more network-like representations to describe the evolutionary relationships among genomes [3, 34–36].

In contrast to prokaryotes, eukaryotes undergo recombination during meiosis and sex, and recombination is always reciprocal [37]. Although eukaryotes are descended from prokaryotes [38, 39], at eukaryote origin they apparently lost the LGT mechanisms typical of prokaryotes, because eukaryotes have so far not been observed to undergo inter-specific (or inter-phylum) conjugation, transformation, or transduction, nor have any genes or proteins been described in eukaryotes that would mediate prokaryotic-type LGT. As a consequence, prokaryotes clearly have pangenomes [12, 28–30], but eukaryotes apparently do not. Neither 1000 human genomes [40] nor 1135 *Arabidopsis* genomes [41] harbored any hint of evidence for the existence of a pangenome or pangenome-like structure. By contrast, the existence of pangenomes in prokaryotes became evident based upon only a handful of sequences per species [12, 28–30]. The only mechanism characterized as a source of new genes entering nuclear genomes in a natural manner is gene transfers from organelles [42]. Barring targeted gene transfer experiments [43] and endosymbiont genome insertions into insect chromosomes with contiguous sequences [44], reports of prokaryote-to-eukaryote LGT are based on sequence comparisons and annotations of individual genes. Thus, in contrast to LGT among prokaryotes, which is their natural mechanism to generate new gene combinations, the role of LGT in eukaryote evolution is controversial.

Some reports suggest that prokaryote-to-eukaryote LGT frequently occurs in phagotrophic, unicellular eukaryotes [45], that there is continuous LGT from prokaryotes to vertebrates and other animals [9] as well as to plants [46] and to algae [47]. In only a few rare and well-documented cases can the sources of LGT to eukaryotes be pinpointed [44, 48], in other cases, the prokaryotic donors are known for their ability to transfer DNA to eukaryotes [49], and of course eukaryotes acquired many genes from the endosymbiotic ancestors of mitochondria and chloroplasts [50]. Yet for the vast majority of cases reported for prokaryote-to-eukaryote LGT, the mechanisms and specifics (how, when, and between which groups) remain obscure.

If the numerous claims for eukaryotes constantly acquiring prokaryotic genes through LGT [51–58] are true, then there would indeed seem to be no natural barrier for prokaryote-to-eukaryote LGT. That leads to two important questions: (1) If such claims are true, what are the implications for our understanding of evolution? But that is not our question here, rather we ask the second question: (2) Are such claims true? Importantly, asking whether eukaryotes are constantly acquiring genes from prokaryotes is not the same as asking if prokaryote-to-eukaryote LGT *never ever* occurs. After all, examples like the genome fragments that are present in insect genomes and that were acquired from bacterial endosymbionts of the insect lineage [44, 48] or *Agrobacterium* colonization in plants [49] show that sometimes genes do make their way from prokaryotes to eukaryotes. We are thus not going to ask whether the barriers to gene flux from prokaryotes and eukaryotes are absolute and have *never* been crossed during evolution, because we already know that they have, in particular at the origin of chloroplasts and mitochondria [50]. Rather we are going to ask whether prokaryotic genes enter the eukaryotic lineage at a frequency that has detectable evolutionary impact and leaves clear evidence in the form of genes in eukaryotic genomes that were recently acquired from prokaryotes.

In previous work, we showed that acquisitions of prokaryotic genes by the eukaryotic lineage correspond to endosymbiotic events (the origins of mitochondria and chloroplasts) [50] and that many of the patterns of "patchy" gene distributions that some reports interpret as evidence for LGT [51, 52] are in fact more likely the result of differential loss [50] superimposed upon vertical inheritance. Those findings are not compatible with claims that eukaryotes are constantly and frequently acquiring genes from prokaryotes. Something has to give.

How to test claims for abundant LGT from prokaryotes to eukaryotes? If LGTs from prokaryotes to eukaryotes are as commonplace and as frequent as many papers assert [4, 9, 10, 45, 51–58], then eukaryote genomes should contain both anciently acquired prokaryotic genes and recently acquired prokaryotic genes. Furthermore, it should be possible, using robust measures, to uncover evidence for the presence of recently acquired genes. Here we look for recent LGTs from prokaryotic donors in eukaryotic genomes and — for direct comparison to a positive control where recent LGTs should be detectable — in prokaryotic genomes as well.

## Results

The essence of our approach is simple: Recent LGT in prokaryotes deposits new donor sequences in recipient genomes that show very high sequence identity between donor and recipient lineages [2, 59]. The high sequence identity between donor and recipient (initially 100 %) gradually deteriorates over time because of mutation (amelioration) so that more recent transfers tend to show higher similarity to homologs from the donor lineage [32, 60]. Thus, if 5.1 % [52] or even 16.1 % [7] of the genes in a given eukaryote come from prokaryotes via constant LGT accumulation over time [46, 51, 54, 55, 61], eukaryote genomes should exhibit distributions of donor-recipient sequence identity (ancient and recent transfers) comparable to those seen in prokaryotes. If not, something is wrong with the eukaryote LGT reports. That can be tested with genome data.

The present data comprise 2585 phylogenetic trees from clusters (alignments) that contain homologs from prokaryotes and eukaryotes, also designated as eukaryotic-prokaryotic clusters (EPCs) [50]. Each of these clusters, generated from 55 eukaryotic and 1981 prokaryotic genomes (Additional file 1: Table S1), contains at least two eukaryotic and at least five prokaryotic sequences, and the sequence similarity threshold for clustering is on the order of ≥25 % in pairwise comparisons [50]. The criterion of requiring genes to be present in at least two eukaryotic genomes serves to eliminate obvious bacterial contaminations from the data. Yet, as we will see, the two-eukaryote-genome criterion does not remove contaminations that are less obvious. The criterion of having at least five prokaryotic sequences in the cluster is to provide a reference tree framework for the investigation. The 25 % amino acid sequence identity criterion is stricter than that employed in many other protein cluster databases, such as the Clusters of Orthologous Groups (COG) [62] or EuKaryotic Orthologous Groups (KOG) [63] databases. Our clusters are generated for the purpose of generating alignments and phylogenetic trees, whereby pairwise sequence identity at or below 20 % leads to problematic alignment and problematic trees [64].

In trees generated from the COG databases, for example, more than 40 % of trees exhibit what was once called ”pseudoparalogy”; that is, the clusters unite several very distantly related prokaryotic and eukaryotic gene families into the same tree [65]. This is fine if functional annotation is the goal (a main goal of many such databases), but problematic if alignments and trees are the objective of investigation. For the present data spanning 2585 trees in which all sequences are uniquely assigned (no sequence occurs in more than one cluster), the number of taxa in each cluster is shown (Additional file 2: Figure S1), the mean number of eukaryote taxa per tree is 10.6, and the mean number of prokaryote taxa per tree is 247.

**Fig. 1:**
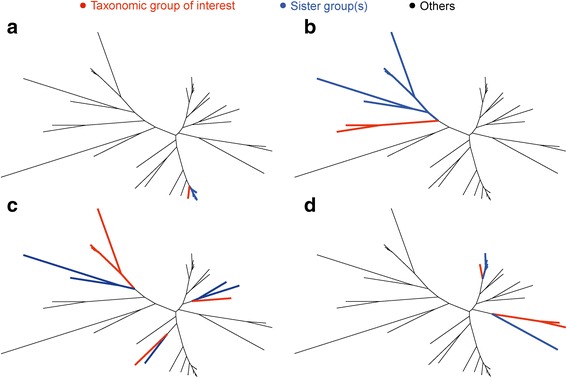
Identification of clades and sister groups. For each tree, largest possible clade(s) and their respective sister group(s) are identified for different taxonomic groups (e.g., eukaryotes or bacilli). One (**a**, **b**) or more (**c**, **d**) clades can be present for a single taxonomic group, with close (**a**), divergent (**b**, **c**), or both close and divergent sister groups

The simplest way to look for evidence of recent transfer is to compare sequences from a clade of a given taxonomic group (for example, eukaryotes or bacilli) to the sister group of that clade in a maximum likelihood tree (Fig. 1). For recent transfers, the proportion of identical amino acid residues for the clade-sister comparison (*I*_C-S_) should be high, up to 1.0 (100 % amino acid identity) for very recent acquisitions from outside the taxon (Fig. 1a). For more ancient transfers (Fig. 1b, c), values of *I*_C-S_ should be lower, with a lower bound near 0.25 because of the 25 %-identity clustering threshold [50]. A taxonomic group can have more than one clade in a tree (Fig. 1c, d), and both recent and ancient transfers can be observed in the same tree (Fig. 1d).

**Fig. 2:**
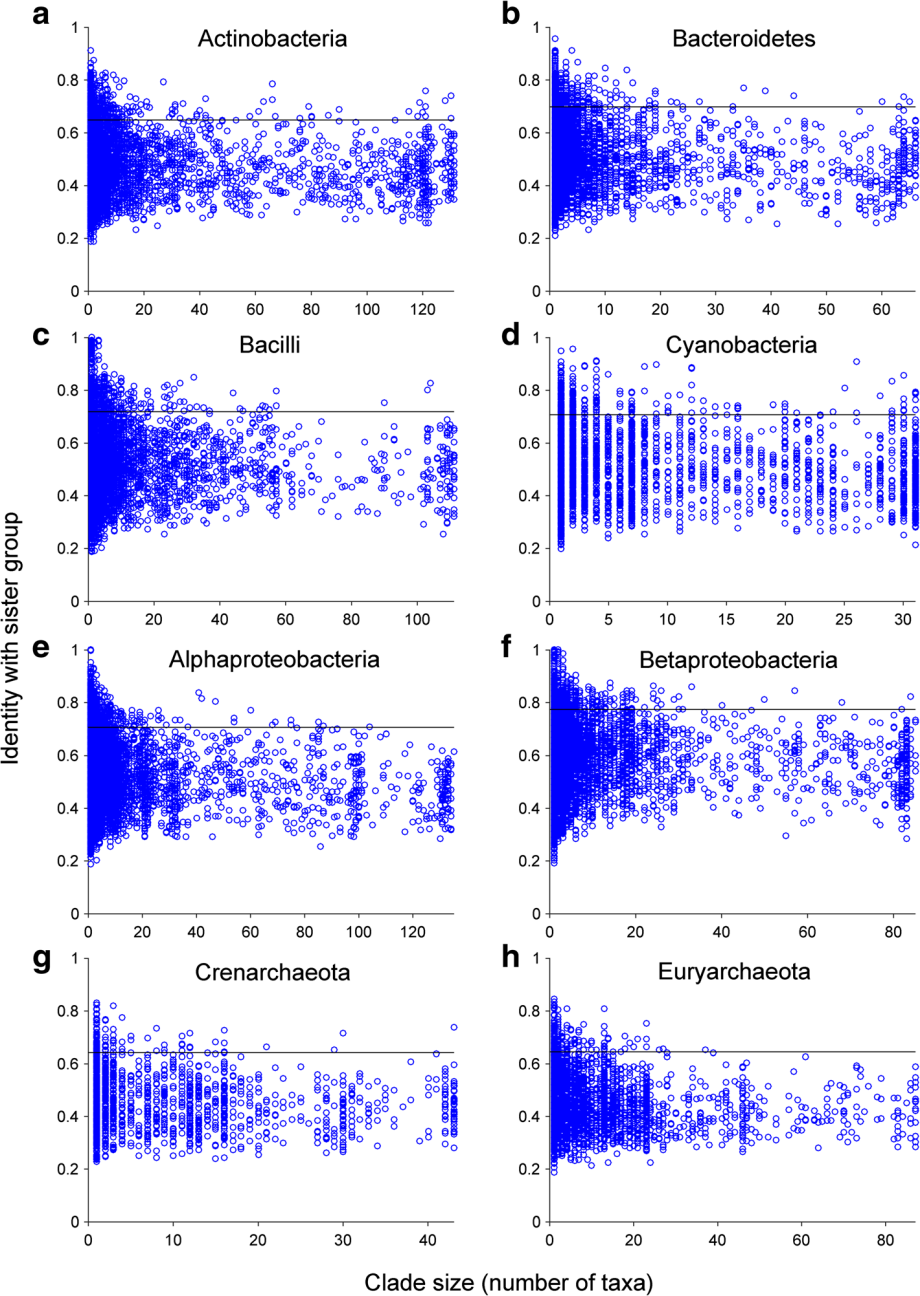
Phylogenomic dissection of major prokaryotic groups. All largest possible clades are plotted for each taxonomic group. *y-axis*: average sequence identity between a clade and its sister group (*I*_C-S_); *x-axis*: number of taxa (species in bacteria or genomes in archaea). A *horizontal reference line* is drawn corresponding to the average of the singleton *I*_C-S_ greater than or equal to their third quartile. **a**–**f** Bacterial groups. **g**–**h** Archaeal groups

**Fig. 3:**
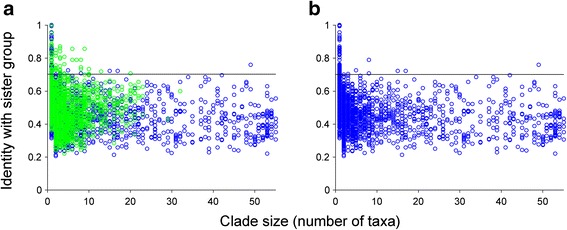
Phylogenomic dissection of eukaryotes. All largest possible eukaryotic clades are plotted. *y-axis*: average sequence identity between a clade and its sister group (*I*_C-S_); *x-axis*: number of species. A *horizontal reference line* is drawn corresponding to the average of the singleton *I*_C-S_ greater than or equal to their third quartile. **a** All clades. **b** Clades of plastid origin (shown in *green* in **a**) are selectively removed

For prokaryotic groups (Fig. 2; Additional file 3: Figure S2) and for eukaryotes (Fig. 3), we plotted all values *I*_C-S_ (*y*-axis) that could be extracted from the 2585 trees against the number of taxa (*x*-axis) in the clade for each comparison. For bacilli, α-proteobacteria, and β-proteobacteria, we observed very recent transfers in the form of *I*_C-S_ values of 1.00 (complete identity to the sister group), whereas for the two archaeal groups (Crenarchaeota and Euryarchaeota), the highest *I*_C-S_ is only approaching 0.85. To compare quantitatively the relative frequency of high *I*_C-S_ values, the singleton clades (i.e., only one taxon) in the respective taxonomic group were used as the reference. For each taxonomic group, a reference value was used as the lower bound of the high sequence identity characteristic of recent LGTs, which was calculated as the average of the singleton *I*_C-S_ that are greater than or equal to their third quartile (Additional file 4: Table S2). If the *I*_C-S_ of a clade is greater than or equal to this reference value, it is then a high-identity clade (HIC). All prokaryote groups exhibited numerous *I*_C-S_ values above their reference line (Fig. 2a–h; non-singleton HICs comprise 3.1–5.1 % of all clades).

### Deep differences between prokaryotes and eukaryotes

A list of the functional annotations for the top ten clusters with the most conserved (most recent) acquisitions for each panel in Figs. 2 and 3 is given in Additional file 5: Table S3. These recently transferred genes encompass mainly metabolic functions, which is in line with the view that prokaryotes generate diversity mainly through acquisition, rather than through duplication [31].

By contrast, values of *I*_C-S_ for eukaryotes were rarely above the reference value 0.70 (Fig. 3; non-singleton HICs comprise < 1.0 % of all clades and 0.3 % in Fig. 3b). Two aspects of the eukaryote comparisons are particularly noteworthy. First, in Fig. 3a the points plotted in green indicate *I*_C-S_ values for genes of plastid origin (clusters in blocks a–c in Figure 1 of [50] and other clades consisting of taxa only from Archaeplastida, from Archaeplastida and SAR or Hacrobia, or from all three). The green points for *I*_C-S_ above the reference value could in principle correspond to recent transfers, yet if we look at the functions involved (Additional file 6: Table S4), they are mainly plastid-related, such as phycobiliproteins, components of the extrinsic photosynthetic antenna complex found in some of the algal lineages. These are not recent acquisitions; rather they were acquired from cyanobacteria at the origin of primary plastids, as earlier investigations have shown [66]. Their high *I*_C-S_ values reflect unusually high sequence conservation, not recent acquisition.

If we plot only the eukaryotic *I*_C-S_ values for clades not of plastid origin (Fig. 3b), a very remarkable pattern comes to the fore in that only eight non-singleton HICs remain, including the clade E211_B160_0 (49 species; identity 0.76) of the ATP synthase subunit beta and E2540_B5394_A3181_1 (8 species; identity 0.79). The latter corresponds to a sea anemone *Nematostella* sequence (jgi|Nemve1|78454|gw.12527.1.1) nested within a clade otherwise specific to photosynthetic eukaryotes, which is probably a contamination (see below).

After the removal of clades of plastid origin, there are 69 singleton HICs. By singleton we do not mean proteins present only in one eukaryotic genome, because each tree has sequences from at least two eukaryotic genomes. Rather, singleton means that only one eukaryotic taxon is in the clade, separated from the other eukaryotic clade(s) in the tree. The identity and functional annotation of these eukaryotic singletons reveal that they mostly stem from the *Nematostella* and *Amphimedon* genome sequences. The genome sequence of *Nematostella* has an unexpectedly large number of predicted protein domains [67] and is known to contain many contaminating sequences from bacteria [68], which also seems to be the case for the genome sequence of the sponge *Amphimedon* [69].

**Fig. 4:**
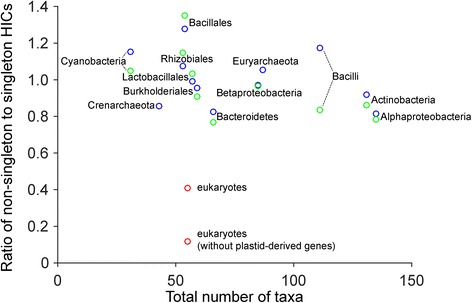
Eukaryotes have relatively fewer non-singleton high-identity clades (*HICs*). Taxonomic groups are plotted according to their ratio of non-singleton HICs to singleton HICs against their number of taxa. *Red*: eukaryotes with all clades (Fig. 3a) or with clades of plastid origin removed (Fig. 3b); *blue*: prokaryotic groups based on the original eukaryotic-prokaryotic clusters (Fig. 2; Additional file 3: Figure S2); *green*: prokaryotic groups based on clusters generated using the same clustering procedure as for eukaryotes (Additional file 8: Figure S3)

That the singletons in the eukaryotic comparisons represent an anomaly is reflected in two further ways. First, if we plot the ratio of non-singleton to singleton HICs (Fig. 4; Additional file 4: Table S2), the eukaryotes stand out and are significantly different from the prokaryotes at *p* < 0.01 for all clades or *p* < 1 × 10^−6^ when clades of plastid origin are removed (standard Pearson chi-square test; Additional file 7: Table S5). One factor that may influence the numbers of non-singleton and singleton HICs is the different clustering procedures for eukaryotes and prokaryotes [50], especially the different global identity cutoff for sequence pairs to be clustered (40 % for eukaryotes and 25 % for bacteria or archaea). This could result in a lower reference value in prokaryotes and might influence the ratio. To test this effect, we redid the analyses by clustering sequences of each bacterial group using the procedure for eukaryotes (see Methods). After the reanalyses (Additional file 8: Figure S3), prokaryotes are still significantly different from eukaryotes at *p* < 0.01 for all clades or *p* < 1 × 10^−6^ for clades of non-plastid origin (Additional file 7: Table S5).

**Fig. 5:**
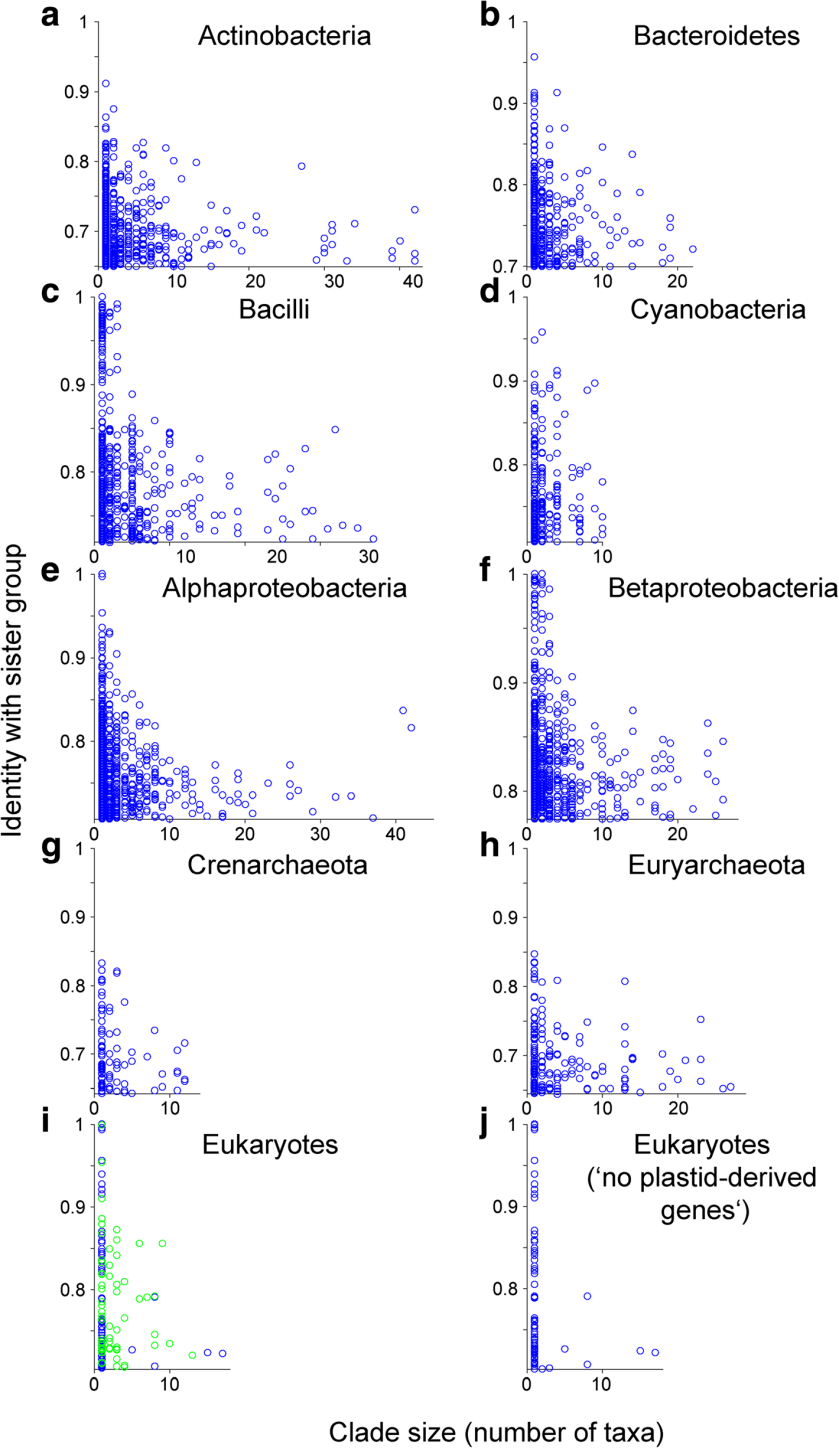
Close-up of the distribution of small-sized high-identity clades (*HICs*). HICs with up to one-third of the total taxa are shown for each group in Figs. 2 and 3 (with *x-axis* plotted to the same scale for each group). **a**–**h** Prokaryotic groups. **i** All eukaryote clades. **j** Eukaryotes with clades of plastid origin (shown in *green* in **i**) selectively removed. The seven proteins having >70 % sequence identity to prokaryotic homologs but appearing in more than one eukaryotic genome are annotated as (*from left to right*): fructose-bisphosphate aldolase, unknown (carbohydrate transport and metabolism), homocitrate synthase, component of cytochrome b6f complex, ribulose-phosphate 3-epimerase, pyridoxal biosynthesis, and adenosylhomocysteinase

**Fig. 6:**
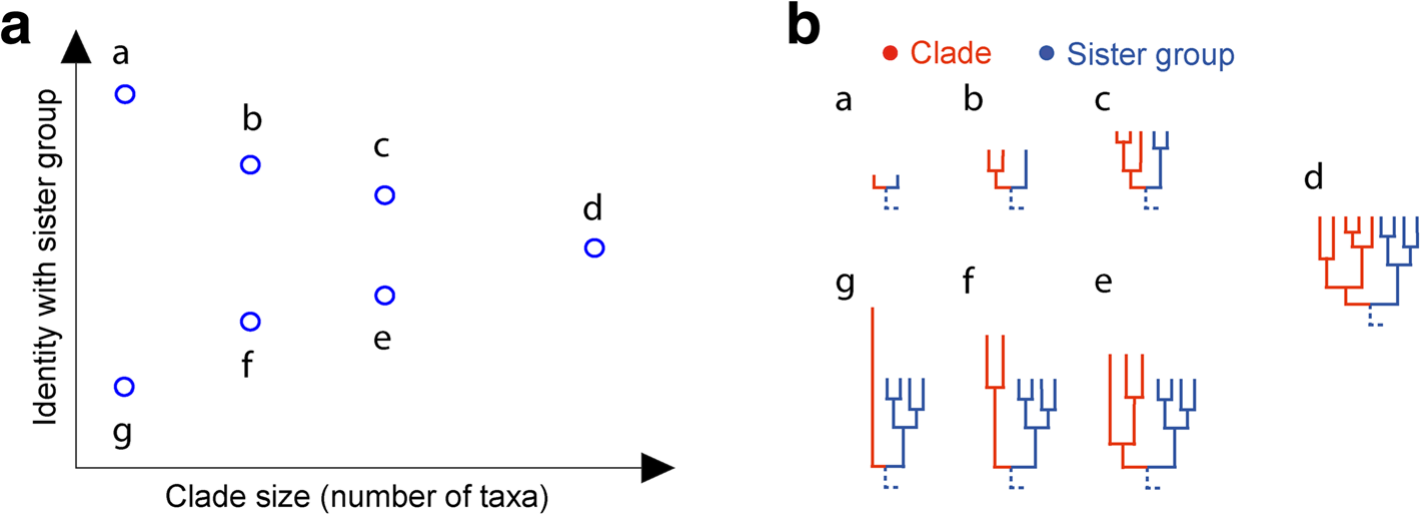
Distribution of clades in the phylogenomic space. **a** Seven representative clades are plotted in the phylogenomic space with clade-sister identity as the *y-axis* and clade size as the *x-axis*. **b** Phylogenetic trees corresponding to the seven clades illustrate the effects of lineage diversification (*a*–*d*), sequence divergence (*a*–*g*), and differential gene loss (*d*–*g*)

Second, if we zoom in on HICs that are up to one-third of the total taxa in size (Fig. 5), we see that the prokaryotic acquisitions show a normal and expected tendency to become less similar to their sister group, the more taxa there are in the clade in question. In other words, genes acquired by prokaryotes can be transmitted vertically in the new lineage, and as they do so, they accumulate sequence divergence relative to the sister group, while at the same time lineage diversification takes place, such that the new gene is present in increasingly many descendant lineages (Fig. 6). What we see in Fig. 5 is basically a snapshot of continuous pangenome formation in prokaryotes, while in eukaryotes nothing of the sort is observed.

That HICs of non-plastid origin are mainly restricted to singletons can mean one of two things. It may suggest that eukaryotes do undergo lateral gene acquisition from prokaryotes, but that the acquisitions are very short-lived and do not persist to the lineage diversification stage, in which case they have no evolutionary significance at all. The more likely alternative is, however, that the singletons showing more than 70 % amino acid identity (reference value in Fig. 3) to their closest prokaryotic homolog are simply contaminations that during genome annotation procedures were scored as similar enough to eukaryotic homologs to represent a bona fide eukaryotic gene to be included in the assembly. The 70 % amino acid identity threshold seems to be the result of a natural inter-domain barrier to LGT between prokaryotes and eukaryotes. Eukaryotic sequences that share ≥70 % amino acid identity to prokaryotic homologs are probably not lateral gene transfers at all, but just contaminants.

## Discussion

In the present paper, we are asking a fairly simple but very controversial question: Are the many highly publicized claims for LGT from prokaryotes to eukaryotes real, or are they artifacts stemming from some combination of (1) genome sequencing contaminations, (2) annotation practice, (3) phylogenetic reconstruction, (4) the underappreciated role of differential gene loss in eukaryote genome evolution, or (5) a combination of the above? Microbiologists have long known about the existence of LGT among prokaryotes [13] and furthermore anticipated the existence of pangenomes in that they built up to 30 % difference in gene content into the prokaryote species definition [70]. Genome sequences, however, have uncovered an extent of LGT among prokaryotes that no one really anticipated. For example, the current estimates for the pangenome size of a single species, *Escherichia coli*, based on 2085 sequenced strains, are now at 90,000 genes and still climbing, linearly [71]. No mechanism other than LGT will produce pangenomes of that size, and the basic concept of LGT among prokaryotes has never been controversial, because it is a natural process and meshes well with what we know about prokaryote biology.

So if we look back to 1998, when the first evidence for substantial LGT from genome sequence analyses was emerging [59], we can now be absolutely certain: Yes, there can be no doubt that LGT in prokaryotes is real, that it is ongoing, and that it reflects a very important aspect of prokaryote biology: natural variation through recombination. At the same time, endosymbiotic theory has always stated that many genes entered the eukaryotic lineage via the endosymbiotic ancestors of mitochondria and chloroplasts; of this we can also be certain [42, 50, 66, 72]. The basic concept of endosymbiotic gene transfer [73] has also never been controversial, because it is a natural process and meshes well with what we know about eukaryote biology.

The aspect of LGT that has been controversial — but perhaps not controversial enough in our view — concerns claims for outright LGT from prokaryotes to eukaryotes outside the context of endosymbiosis. Such claims were put forth in the human genome sequence [4], and they were promptly refuted as artifacts [5, 6]. New claims for prokaryote-to-eukaryote LGT soon emerged, they became popularized by LGT proponents [58], and soon thereafter many or most eukaryotic genome sequences published in high-profile journals contained reports (or claims) for more LGT [7, 54, 55]. Claims for LGT from chlamydiae to the plant lineage [47, 54, 74, 75] have been repeatedly published, but also repeatedly tested and rejected [50, 76–80], and the same claims have been advanced again recently [81], ignoring the many tests [50, 76–80] that refuted such claims, as if LGT claims are somehow immune to scientific testing. Patchy gene distributions in eukaryotes are also often interpreted as evidence for LGT [45], without even considering the alternative: differential loss [50]. The high tide of prokaryote-to-eukaryote LGT claims might have been reached with the tardigrade showdown, where one group reported that 16.1 % of all tardigrade nuclear genes are recent LGTs from prokaryotes [7], while a separate study found almost none at all [8].

If the claims from individual genome sequences for prokaryote-to-eukaryote LGT are real, then it means that eukaryotes have indeed been continuously acquiring genes from prokaryotes over evolutionary time. That in turn predicts that we should then see two fundamental patterns in investigations of eukaryotic genome sequences. First, different lineages of eukaryotes should possess fundamentally different collections of prokaryote-derived genes, just as we see in prokaryotes [11, 12, 30]. Second, eukaryotic genomes should harbor evidence for recently acquired prokaryotic genes, in addition to the anciently acquired genes that entered eukaryote genomes at the origin of mitochondria and plastids.

Few tests of either prediction have been reported. The obvious test for the first prediction (lineage-specific gene acquisitions) is simple: If we investigate gene presence and absence across many different eukaryotic lineages, then genes that eukaryotes share with prokaryotes should reveal patterns of lineage-specific acquisition. But the converse is observed: The only evidence for lineage-specific gene acquisition in eukaryotes is the mass introduction of bacterial genes in the plant lineage corresponding to the origin of plastids and their subsequent spread during secondary symbiosis [50]. Lineage-specific gene losses in eukaryotes are, by contrast, very common [50].

### The 70 % rule

A thorough test of the second prediction (evidence for recent and ancient gene acquisitions) has been lacking. If eukaryotes are acquiring genes from prokaryotes continuously during evolution, then eukaryotic genomes should reveal evidence for recent acquisitions. Here we sought such evidence. We find that prokaryotes do indeed acquire genes from outside their phylum continuously during evolution, while eukaryotes do not. Prokaryotic phyla show a typical pattern of recent acquisitions that show up to 100 % amino acid sequence identity to their sister-group homologs (Fig. 2). The only examples of such high amino acid sequence identity between prokaryotic and eukaryotic genes are restricted to singleton clades, such as E2190_B358_A1066_1 and E2268_B77_0 from *Nematostella* (Additional file 9: Table S6), which is known to harbor many contaminations [68, 82]. There are a few proteins in plastid-bearing eukaryotes that exhibit >80 % amino acid sequence identity to prokaryotic homologs, but these are mostly involved in photosynthetic functions; they are acquisitions that correspond to the origin of plastids (Additional file 6: Table S4).

If we look among the 2386 clades of non-plastid origin, only very few proteins, such as mitochondrial ATPase, an acquisition corresponding to mitochondrial origin, have ≥70 % amino acid sequence identity among proteins present in more than one eukaryotic genome. All other eukaryotic protein sequences showing ≥70 % amino acid sequence identity to prokaryotic homologs are either (1) acquisitions from the plastid ancestor or (2) contaminations. Genes shared by prokaryotes and only one eukaryotic genome are suspects for contamination anyway. In the present study, we have queried 2386 sequence comparisons, such that the paucity or absence of pairwise identity ≥70 % between clades of eukaryotic proteins present in more than one genome and homologs from prokaryotic sister group clades might be rather general. We call it the 70 % rule.

### Sampling and rates?

Critics might wonder about possible effects of uneven sampling in our present investigation. The prokaryotic groups examined have many dozens of species in each case (ranging from 31 to 135; Additional file 4: Table S2), and there are several dozen eukaryotes, too (55 species). Recalling that Fig. 2 shows the results for the comparison of sequences from a given prokaryotic group to the sister group sequence(s) from other taxa, we see a continuum reaching up to >90 % and sometimes 100 % average identity, reflecting continuous recent acquisitions. Compared to *the same* prokaryotic groups, the 55 eukaryotes top out at 70 % — the corresponding evidence for recent LGTs does not exist. Thus, the nature of the comparisons takes the somewhat uneven sampling into account. Critics might also wonder whether genes are constantly flowing from prokaryotes into eukaryotic genomes, but undergoing rapid evolution once they arrive so as to conform to the 70 % rule. That is a special plea, but we can exclude it nonetheless. Were that true, then different groups of eukaryotes would have fundamentally different collections of prokaryotic genes, but that possibility has already been tested and it is not the case: Eukaryotes possess different subsets of one and the same set of prokaryotic genes, which was present in the eukaryote common ancestor [50]. Critics might also offer that the eukaryotic genes are so divergent from their prokaryotic sisters because we do not know (or have not sampled) prokaryotic lineages closely related to the donors. But Fig. 2 shows that for the same sample of genes, we do see the donors in prokaryotes; that is, we find many sequences having >70 % identity to sisters from outside the phylum. Hence the prokaryotic sample cannot be the problem.

### The last one out…

If lineage-specific acquisitions are extremely rare in eukaryotes, as the present data indicate, how can one explain the presence of lineage-specific genes that are present in more than one genome? There are two ways to explain sparse gene distribution patterns: lineage specific acquisition or differential loss. If a gene is lost in one lineage, that means that it cannot be essential, hence it is possible for it to be lost in other lineages as well. Furthermore, loss is an irreversible process — genes lost in one lineage will be missing in all descendants. If genes are indeed undergoing widespread loss in eukaryotes, as recent studies indicate [50, 83], it follows that some genes will have been lost in all lineages but one. Such genes (present only in one group) will have typical eukaryotic attributes, such as normal promoters and introns, and like other eukaryotic genes of prokaryotic origin they will be distantly related to their prokaryotic homologs, but they will be lineage-specific (but not genome-specific, like singleton contaminations).

This is exactly what is observed for genes that were interpreted as evidence for LGT in the *Galdieria sulphuraria* genome [55], a genome with claims for abundant LGT [84]. Whereas Richards and Monier [84] remain receptive to the claim for an LGT origin of 5 % of the genes in *Galdieria* [55], they do not mention the possibility of differential loss to explain this curious gene presence pattern. We consider it likely that those *Galdieria* genes are the result of differential loss in other genomes. After all, if a gene can be lost in one lineage, it can be lost in other lineages as well, and in the last lineage to retain the gene it will look in terms of gene distribution all the world like an LGT, but it will conform to the 70 % rule. In differential loss, the last one out looks like an LGT.

## Conclusion

Here we devised an approach to summarize the effects of LGT in prokaryotic and eukaryotic genome evolution. Our findings indicate that eukaryotes do not acquire genes through continual LGT like prokaryotes do. Major gene acquisitions do occur in eukaryote evolution, but these correspond to endosymbiotic events [50]. By contrast, evolutionarily recent acquisitions from prokaryotes appear to be too rare to have broad evolutionary significance. In prokaryotes, both vertical inheritance and gene acquisition from other prokaryotes via LGT contribute to the distribution of genes across genomes. In eukaryotes, the situation regarding gene acquisitions via LGT has been more controversial. Our present findings support the view that, in eukaryotes, a stem gene repertoire was already present in the complex last eukaryotic common ancestor [67], with endosymbiotic events and differential loss [50] determining the subsequent distribution of genes across eukaryotic genomes.

## Methods

Eukaryotic, archaeal, and bacterial protein sequences were clustered separately and combined into 2585 eukaryotic-prokaryotic clusters (EPCs) using the reciprocal best cluster approach as reported in a previous study [50]. Sequences within each cluster were aligned with Multiple Alignment using Fast Fourier Transform (MAFFT) v7.130 [85], followed by maximum-likelihood tree inference using Randomized Axelerated Maximum Likelihood (RAxML) v7.8.6 [86]. The EPC functional annotations and trees are described in Supplementary Tables 6 and 7, respectively, in [50]. For the purpose of this study, we searched across all the EPC trees for the largest possible clades from a taxonomic group (a clade is a largest possible clade if neither of the two neighboring clades consist only of taxa from that taxonomic group). The prokaryotic groups analyzed include two major archaeal subgroups, Euryarchaeota and Crenarchaeota, as well as Cyanobacteria and Alphaproteobacteria, from which the plastids and mitochondria arose, respectively [50, 87, 88]. In addition, other major bacterial phyla or classes and their large orders with a medium number (50 to 150) of taxa were included. For each largest possible clade, the sister group is defined as the neighboring clade with the smaller average branch distance (i.e., nearest neighbor). For the calculation of *I*_C-S_ values, identities between all pairs of sequences from the clade and the sister group were calculated using the protdist program of the PHYLogeny Inference Package (PHYLIP) v3.695 [89] and averaged. Standard Pearson chi-square tests were implemented using a script in MATLAB R2015a [90].

To test the effect of clustering procedures, new EPCs were generated for each of the ten bacterial groups analyzed. Their sequences were clustered using the same procedure (40 % global identity cutoff; clusters with at least two sequences were retained) for clustering eukaryotic sequences, whereas the sequences from other bacteria were clustered using the original procedure for bacteria (25 % global identity cutoff; clusters with at least five sequences were retained) [50]. These two sets of bacterial clusters were then combined into the complete bacterial set using the reciprocal best cluster approach, before it was combined with eukaryotic and archaeal clusters as for the original EPCs. Alignments and phylogenetic analyses were done for each set of reclustered EPCs as described above.

## Additional files

Additional file 1: Table S1. List of eukaryotic, bacterial, and archaeal taxa. (XLSX 197 kb)
Additional file 2: Figure S1. Number of taxa in eukaryotic-prokaryotic clusters. The 2585 clusters are sorted first by the number of eukaryote taxa (up to 55) and then by the number of prokaryote taxa (up to 1227). See Additional file 1: Table S1 for the list of taxa. (TIF 579 kb)
Additional file 3: Figure S2. Phylogenomic dissection of large prokaryotic orders. All largest possible clades are plotted for each taxonomic group. *y-axis*: average sequence identity between a clade and its sister group (*I*_C-S_); *x-axis*: number of species. A *horizontal reference line* is drawn corresponding to the average of the singleton *I*_C-S_ greater than or equal to their third quartile. (TIF 937 kb)
Additional file 4: Table S2. List of taxonomic groups and numbers of singleton and non-singleton high-identity clades (HICs). (XLSX 43 kb)
Additional file 5: Table S3. Annotations of top ten clusters with highest sequence identities for each taxonomic group. (XLSX 40 kb)
Additional file 6: Table S4. Annotations of eukaryotic high-identity clades of putative plastid origin. (XLSX 42 kb)
Additional file 7: Table S5. The *p* values of chi-square tests comparing the numbers of singleton and non-singleton high-identity clades (HICs) between taxonomic groups. (XLSX 55 kb)
Additional file 8: Figure S3. Phylogenomic dissection of prokaryotic groups based on clusters generated using the same procedure as for eukaryotes. All largest possible clades are plotted for each taxonomic group. *y-axis*: average sequence identity between a clade and its sister group (*I*_C-S_); *x-axis*: number of species. A *horizontal reference line* is drawn corresponding to the average of the singleton *I*_C-S_ greater than or equal to their third quartile. (TIF 2094 kb)
Additional file 9: Table S6. List of clades in Fig. 2, Fig. 3, Additional file 3: Figure S2, and Additional file 8: Figure S3. (XLSX 10406 kb)

## Abbreviations

EPC: Eukaryotic-prokaryotic clusters
HIC: High-identity clades
*I*_C-S_: Proportion of identical amino acid residues for the clade-sister comparison
LGT: Lateral gene transfer
