# Supplementary figures and images for: A natural barrier to lateral gene transfer from prokaryotes to eukaryotes revealed from genomes: the 70 % rule
Source: BMC Biol. 2016 Oct 17;14:89. doi: 10.1186/s12915-016-0315-9 (PMC5067920; doi:10.1186/s12915-016-0315-9)

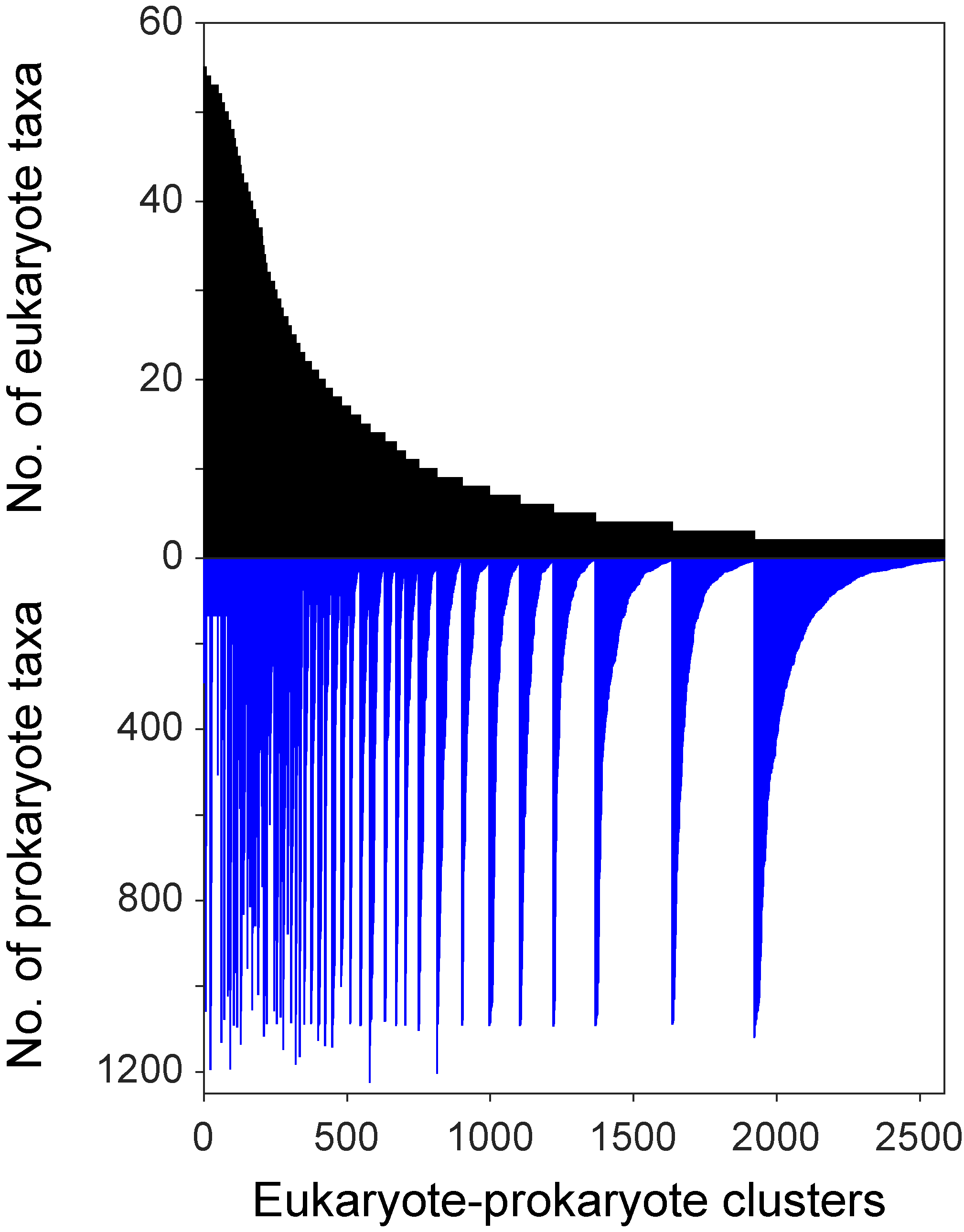

Supplement: Additional file 2: Figure S1. — Number of taxa in eukaryotic-prokaryotic clusters. The 2585 clusters are sorted first by the number of eukaryote taxa (up to 55) and then by the number of prokaryote taxa (up to 1227). See Additional file 1: Table S1 for the list of taxa. (TIF 579 kb) [file 12915_2016_315_MOESM2_ESM.tif]

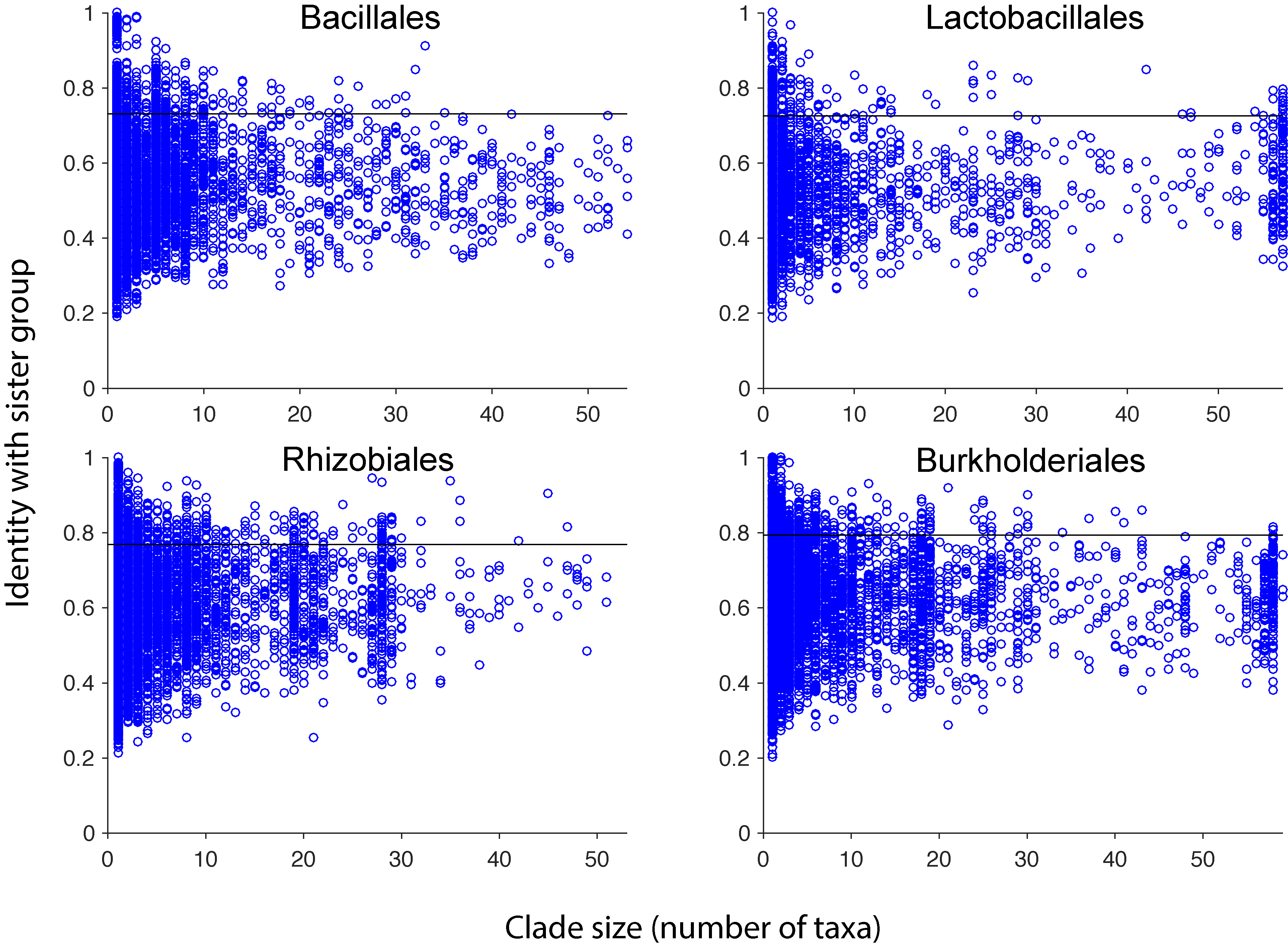

Supplement: Additional file 3: Figure S2. — Phylogenomic dissection of large prokaryotic orders. All largest possible clades are plotted for each taxonomic group. y-axis: average sequence identity between a clade and its sister group (I C-S); x-axis: number of species. A horizontal reference line is drawn corresponding to the average of the singleton I C-S greater than or equal to their third quartile. (TIF 937 kb) [file 12915_2016_315_MOESM3_ESM.tif]

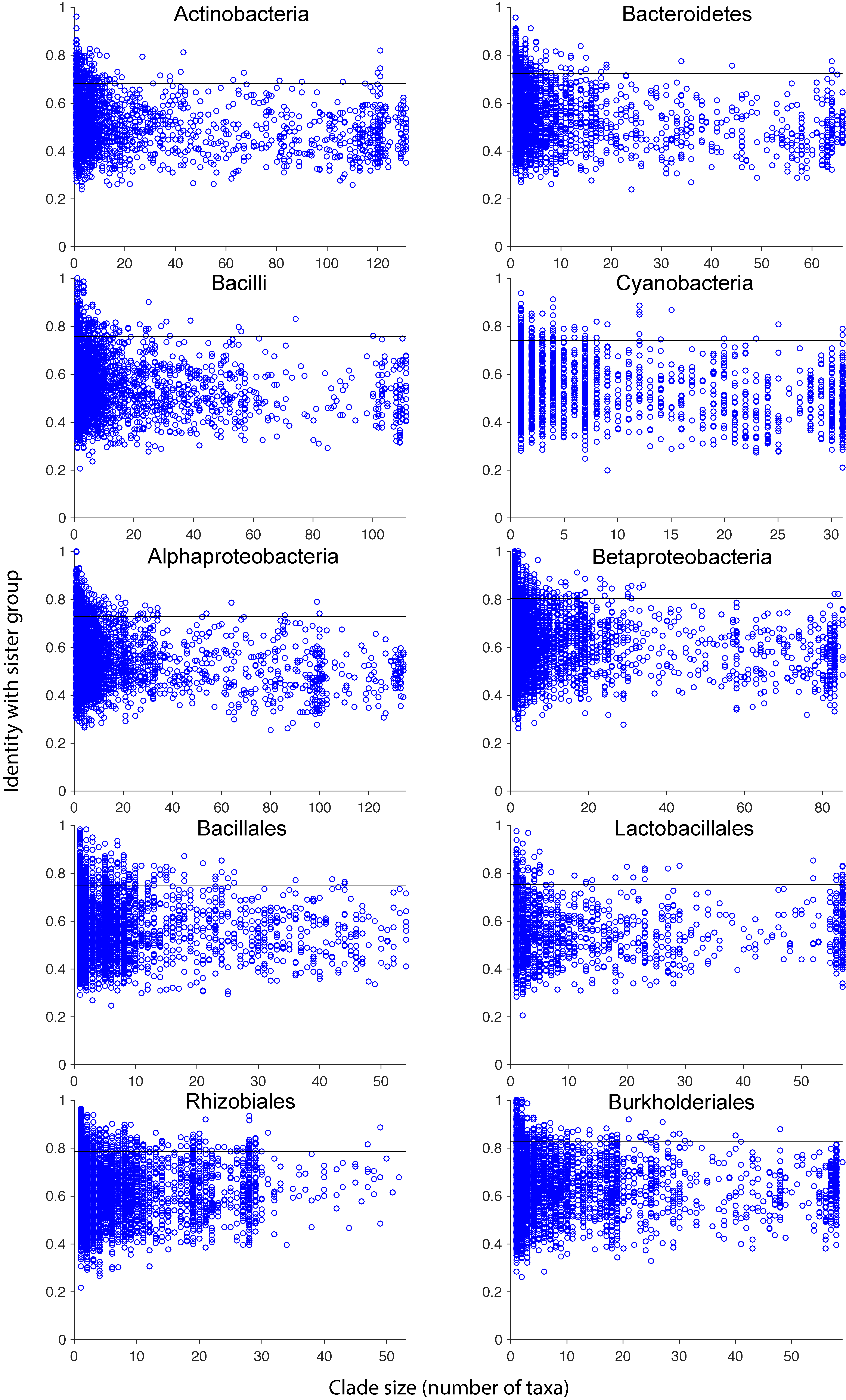

Supplement: Additional file 8: Figure S3. — Phylogenomic dissection of prokaryotic groups based on clusters generated using the same procedure as for eukaryotes. All largest possible clades are plotted for each taxonomic group. y-axis: average sequence identity between a clade and its sister group (I C-S); x-axis: number of species. A horizontal reference line is drawn corresponding to the average of the singleton I C-S greater than or equal to their third quartile. (TIF 2094 kb) [file 12915_2016_315_MOESM8_ESM.tif]
